# α-Halogenated Curcumins Enhance the Binding Affinity and Induced Structural Alterations to Amyloid-β Protein in Alzheimer’s Disease

**DOI:** 10.3390/ijms27209355

**Published:** 2026-10-21

**Authors:** Mateen A. Khan, Raja Chinnappan, Sowmya Andole, Mohd. Akif, Tanveer Ahmad Mir, Ahmed Yaqinuddin, Phuong-T T. Pham, Mamoun M. Bader

**Affiliations:** 1 Department of Life Sciences, College of Science, Alfaisal University, Riyadh 11533, Saudi Arabia; 2 College of Medicine, Alfaisal University, Riyadh 11533, Saudi Arabia; rchinnappan@alfaisal.edu (R.C.); ayaqinuddin@alfaisal.edu (A.Y.); 3 Department of Biochemistry, School of Life Sciences, University of Hyderabad, Hyderabad 500 046, India; andolesowmya@gmail.com (S.A.); akif@uohyd.ac.in (M.A.); 4 Tissue/Organ Bioengineering & BioMEMS Laboratory, Organ Transplant Centre of Excellence (TR & I-Dept), King Faisal Specialist Hospital & Research Centre, Riyadh 11211, Saudi Arabia; tmir@kfshrc.edu.sa; 5 Department of Chemistry, Penn State Scranton, Dunmore, PA 18512, USA; ptp2@psu.edu; 6 Department of Chemistry, College of Science, Alfaisal University, Riyadh 11533, Saudi Arabia; mbader@alfaisal.edu

**Keywords:** Alzheimer’s disease, amyloid-β, α-halogenated curcumin, fluorescence spectroscopy, molecular docking, circular dichroism

## Abstract

Amyloid-β (Aβ) aggregation and associated structural changes are central features of Alzheimer’s disease (AD) pathology. In this study, we investigated the binding of α-halogenated curcumins, α-bromocurcumin and α-chlorocurcumin, to Aβ_1-42_ and examined their effects on the peptide secondary structure using fluorescence spectroscopy and circular dichroism (CD). Both α-halogenated curcumins interacted with Aβ_1-42_, but their binding properties differed from those of the parent curcumin. α-Chlorocurcumin exhibited a higher binding affinity for Aβ_1-42_ than curcumin, whereas α-bromocurcumin showed comparatively weaker binding. Temperature-dependent binding studies revealed that the association of α-halogenated curcumins with Aβ_1-42_ was influenced by temperature, with reduced binding affinity at higher temperatures. Thermodynamic analysis demonstrated favorable free-energy changes for the interactions and revealed that α-halogenation altered the relative enthalpic and entropic contributions to Aβ_1-42_ binding. CD analysis further showed that binding of the curcumin altered the secondary structure of Aβ_1-42_. These structural changes were more pronounced in the presence of the α-halogenated derivatives, indicating that halogen substitution influences the interaction and conformational response of Aβ_1-42_. Molecular docking analysis provided additional insight into the possible binding modes and predicted intermolecular contacts between the α-halogenated curcumin and Aβ_1-42_. Overall, the findings demonstrate that α-halogenation modulates the binding and structural effects of curcumin derivatives on Aβ_1-42_, with α-chlorocurcumin exhibiting particularly favorable binding characteristics. These findings provide a basis for further investigation of α-halogenated curcumins as potential modulators of Aβ-related molecular processes associated with AD.

## 1. Introduction

Alzheimer’s disease (AD) is the most prevalent neurodegenerative disorder in older adults and is characterized by progressive deterioration of cognitive and functional abilities. As the disease progresses, profound memory loss and cognitive impairment increasingly compromise independence and daily functioning, ultimately resulting in substantial loss of autonomy and self-care capacity [1]. A major pathological hallmark of Alzheimer’s disease is the abnormal deposition of amyloid-β (Aβ) peptides in the extracellular space, together with the intracellular accumulation of aberrantly phosphorylated tau protein, giving rise to amyloid plaques and neurofibrillary tangles (NFTs), respectively [2,3]. Among these, Aβ_1-42_, a 42-amino-acid peptide generated through sequential cleavage of amyloid precursor protein (APP) by β- and γ-secretases, has a greater propensity to aggregate than shorter Aβ species. Its aggregation can produce soluble oligomers and subsequently larger fibrillar assemblies that contribute to extracellular amyloid plaque formation. However, Aβ_1-42_ aggregation and plaque deposition represent distinct aspects of amyloid pathology, and soluble Aβ oligomers [4] through the self-association of Aβ peptides, may also contribute to neuronal dysfunction and disease pathogenesis [5,6]. In addition to Aβ and tau pathology, AD involves several interconnected pathological mechanisms, including impaired cerebral blood flow and blood–brain barrier integrity, excessive generation of reactive oxygen species and consequent oxidative damage, chronic activation of microglia and astrocytes with sustained release of pro-inflammatory mediators, and mitochondrial dysfunction characterized by disrupted energy metabolism, impaired calcium homeostasis, and increased oxidative stress. These processes may interact with Aβ accumulation and tau pathology, thereby contributing to synaptic dysfunction, neuronal injury, and disease progression [7].

Curcumin, a naturally occurring polyphenolic constituent of the rhizome of *Curcuma longa* (turmeric), has been widely investigated for its diverse pharmacological properties, including antioxidant, anti-inflammatory, and potential neuroprotective properties [8,9,10,11]. Additionally, curcumin has been demonstrated to have an anti-angiogenic effect by inhibiting the NF-κB pathway [12] and mitigating lipid peroxidation, primarily by diminishing the levels of malondialdehyde (MDA), nitrite, and acetylcholinesterase (AChE) [13]. Most notably, curcumin has also been demonstrated to inhibit the aggregation of Aβ peptide in vitro and in vivo by reducing the β-sheet content in amyloid peptides and disaggregate amyloid fibrils [14,15], indicating a potential protective effect against AD [16,17]. Aβ peptides can aggregate into soluble oligomers, protofibrils, and mature amyloid fibrils, although the relative contributions of different Aβ assemblies to disease progression remain incompletely understood [18]. Given the association between β-sheet enrichment, Aβ oligomerization, and amyloid fibril formation, small molecules that interact with Aβ_1-42_ may offer a potential approach to modulating its aggregation behavior. In this context, curcumin, and its α-halogenated derivatives, α-bromocurcumin and α-chlorocurcumin, are of interest because structural modifications may influence their interactions with Aβ_1-42_ and its conformational properties.

It has been shown that curcumin exhibits a range of biological and medicinal properties [19] to improve mental illnesses due to its ability to modulate numerous signaling molecules [20,21]. The aggregation of Aβ-peptides, particularly Aβ_1-42_, is a central feature of AD pathology. Curcumin has demonstrated the ability to bind Aβ_1-42_ [22], providing a rationale for exploring structurally modified analogs with potentially altered binding properties. A-halogenation may influence molecular interactions and conformational behavior, making α-bromocurcumin and α-chlorocurcumin relevant candidates for comparative investigation. Characterizing the interaction between α-halogenated curcumin and Aβ_1-42_ may help establish structure–activity relationships and identify compounds warranting further evaluation as modulators of amyloid pathology. Curcumin has attracted interest as a potential modulator of Aβ aggregation owing to its role in amyloid assemblies [23]. Previous in vivo studies have reported that curcumin may reduce Aβ aggregation and amyloid deposition, suggesting that its interactions with Aβ peptides warrant further investigation. Accordingly, the present study compares curcumin with its α-halogenated derivatives to assess their binding for Aβ_1-42_ and their effects on peptide secondary structure. These findings may help elucidate how structural modifications influence compound–peptide interactions and provide a basis for further investigation of their potential effects on Aβ aggregation [24,25]. Several reports have been shown the conformational behavior of Aβ in the presence of curcumin and its natural derivatives [14]. These studies revealed that curcumin and its derivatives to interact with Aβ_1-42_ must assume a partially folded conformation with the α-helix form of Aβ_1-42_ and prevent the formation of aggregates [26].

**Figure 1:**
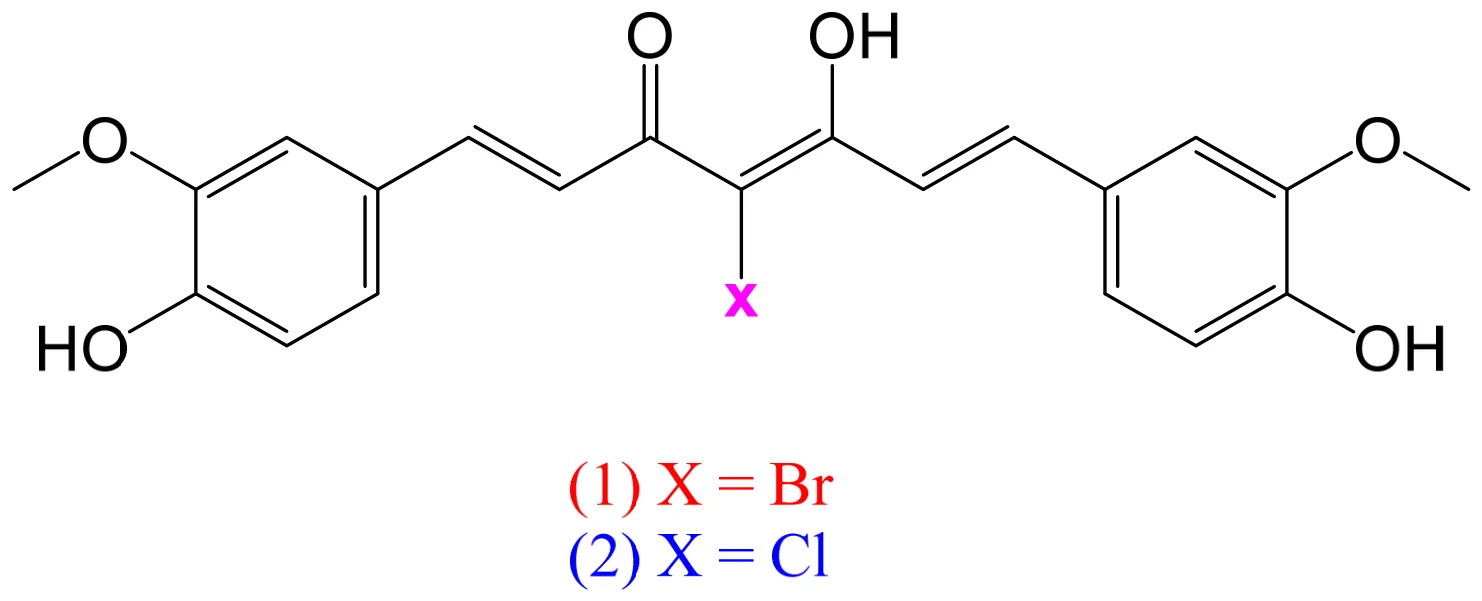
Structures of the α-halogenated curcumin: α-bromocurcumin and α-chlorocurcumin molecules.

Recently Pham and Bader [27,28] synthesized and structurally characterize by single-crystal X-ray diffraction, UV–visible spectroscopy, density functional theory (DFT) calculations, and Hirshfeld surface analysis, the two α-halogenated curcumins: α-bromocurcumin and α-chlorocurcumin with approximately the same solubility in water as the parent compound. Thermal analysis demonstrated that α-bromocurcumin and α-chlorocurcumin exhibited greater thermal stability than unsubstituted curcumin. Furthermore, α-position halogen substitution was associated with increased molecular planarity and shorter π–π stacking distances compared with previously reported curcumin derivatives (Figure 1). Previous studies [29,30] have shown the binding properties of curcumin with Aβ. However, the quantitative binding of α-halogenated curcumins: α-bromocurcumin and α-chlorocurcumin with Aβ_1-42_ and the resultant structural alterations in the protein are still unknown, even though numerous studies relate curcumin’s role in Aβ aggregation.

This study investigated the interactions of α-halogenated curcumin derivatives with Aβ_1-42_ and explored the molecular basis of their binding. Fluorescence spectroscopy and molecular docking were employed to characterize α-halogenated curcumin–Aβ peptide interactions, while circular dichroism (CD) spectroscopy was used to assess associated changes in peptide secondary structure. The results indicated that the α-halogenated derivatives interact with Aβ_1-42_, with binding accompanied by changes in its conformational characteristics and thermodynamic parameters. These findings provide further insight into the binding behavior of α-halogenated curcumin derivatives and their potential influence on Aβ_1-42_ structure.

## 2. Results

### 2.1. Equilibrium Binding of α-Halogenated Curcumins with Aβ_1-42_

**Figure 2:**
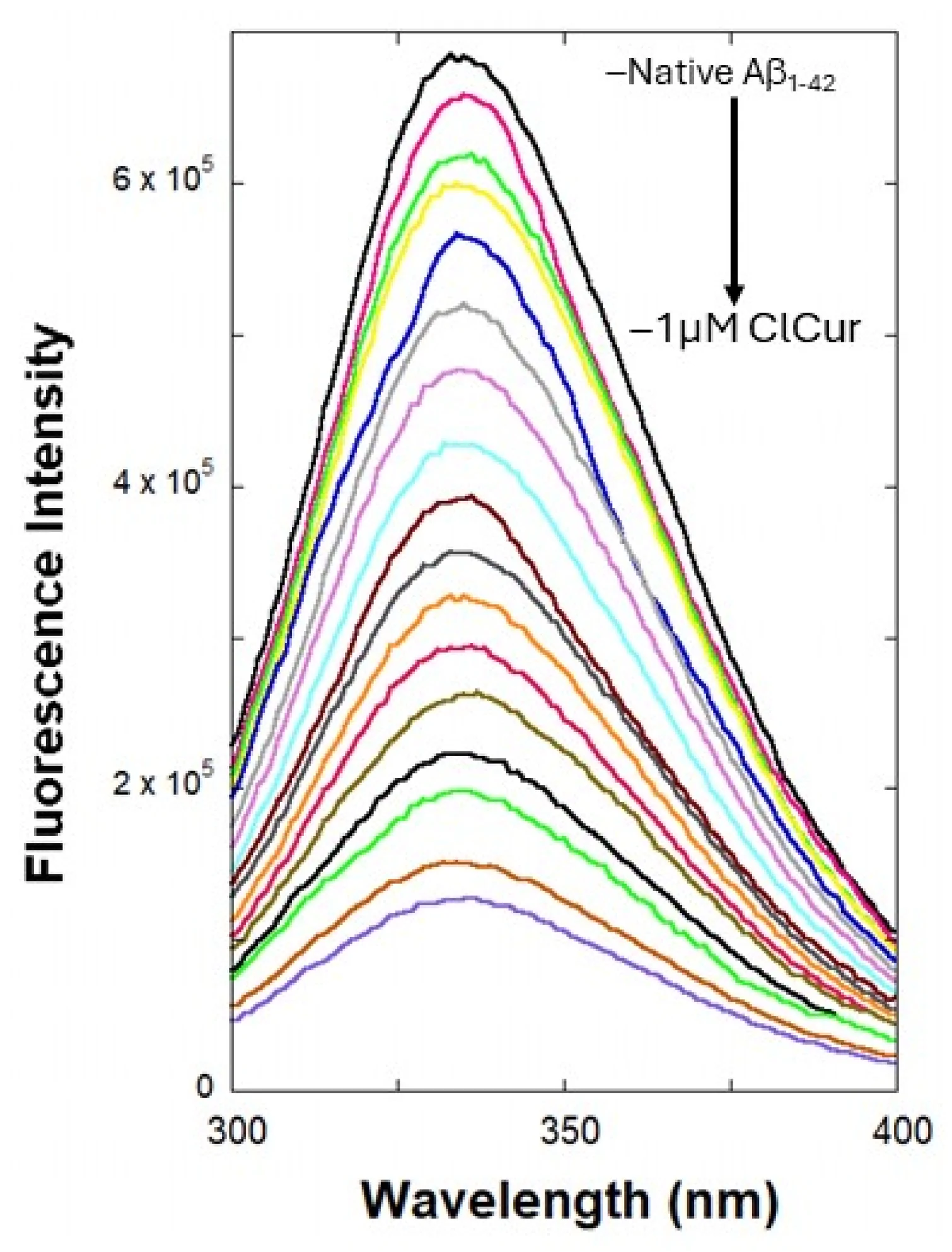
Fluorescence emission spectra of Aβ_1-42_ (0.1 μM) with increasing α-chlorocurcumin concentrations at 25 °C. α-chlorocurcumin-to-Aβ_1-42_ molar ratios ranged from 0 to 10, corresponding to the uppermost and lowermost spectra, respectively. Emission was recorded over 300–400 nm following excitation at 280 nm.

The association of α-bromocurcumin and α-chlorocurcumin with Aβ_1-42_ was examined by monitoring peptide fluorescence across increasing α-halogenated curcumin-to-Aβ_1-42_ peptide molar ratios. Aβ_1-42_ was maintained at 0.1 μM while the α-halogenated curcumin concentration was progressively increased. Following excitation at 280 nm, emission spectra were recorded over 300–400 nm, with an emission maximum at 332 nm. The addition of α-chlorocurcumin progressively reduced the fluorescence intensity at this wavelength, as illustrated by the representative spectra in Figure 2. Curcumin and α-bromocurcumin also produced concentration-dependent decreases in fluorescence under identical experimental conditions.

**Figure 3:**
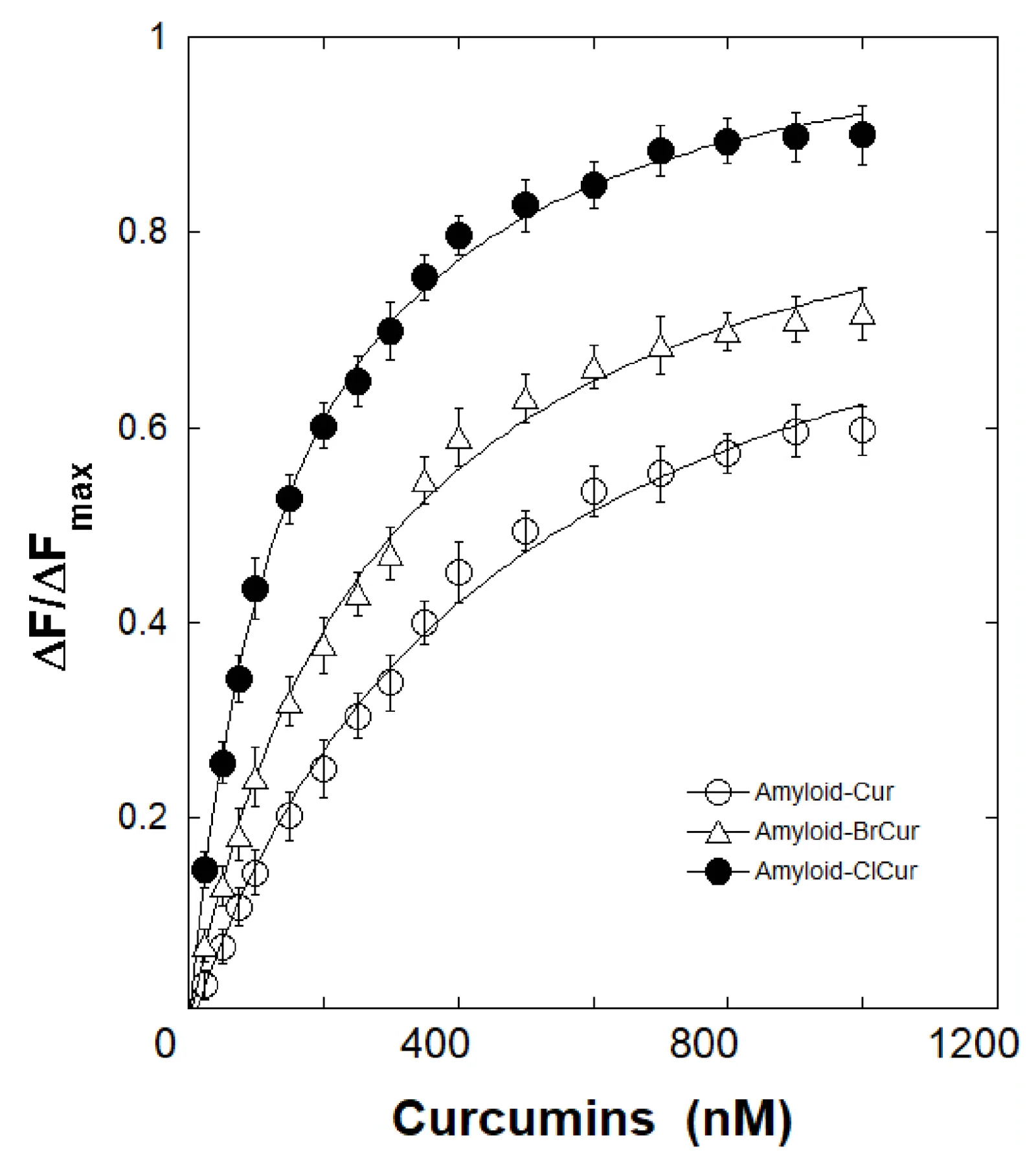
The binding plot for the fluorescence data of Aβ_1-42_ with α-halogenated curcumin derivatives at 25 °C. Fluorescence intensity measurements of Aβ_1-42_-curcumin (―○―), Aβ_1-42_-α-bromocurcumin (−−Δ−−), and Aβ_1-42_-α-chlorocurcumin (―●―) binding. During peptide titration, fluorescence was measured at emission maximum of 332 nm using an excitation wavelength of 280 nm. The data are the average of three independent experiments, and the average value of the data is reported as mean ± SD.

Fluorescence changes at 332 nm were plotted against the α-halogenated curcumin-to-Aβ_1-42_ molar ratio to generate the titration curves shown in Figure 3. At the highest ratios examined, fluorescence quenching reached 55% for curcumin, 72% for α-bromocurcumin, and 90% for α-chlorocurcumin. Thus, α-chlorocurcumin produced the greatest reduction in peptide fluorescence, followed by α-bromocurcumin and curcumin. These observations are consistent with α-halogenated curcumin-associated changes in the fluorescence environment of Aβ_1-42_. However, quenching magnitude alone does not establish relative binding affinity or demonstrate peptide folding or conformational changes; these interpretations require support from binding analysis and complementary structural measurements.

Figure 3 presents the normalized fluorescence response of Aβ_1-42_ to increasing concentrations of α-halogenated curcumins. The progressive decrease in fluorescence is consistent with ligand–peptide association, although quenching alone does not directly quantify the amount of bound ligand. α-halogenated curcumin strongly interacts with Aβ_1-42_. Values of dissociation constant (*K*_d_), obtained from the nonlinear analysis of the fluorescence data, were found to be 524 ± 21 nM for curcumin, 441 ± 16 nM for α-bromocurcumin, and 285 ± 15 nM for α-chlorocurcumin, respectively, which indicates a relatively stronger binding affinity for α-chlorocurcumin as compared to curcumin alone.

**Figure 4:**
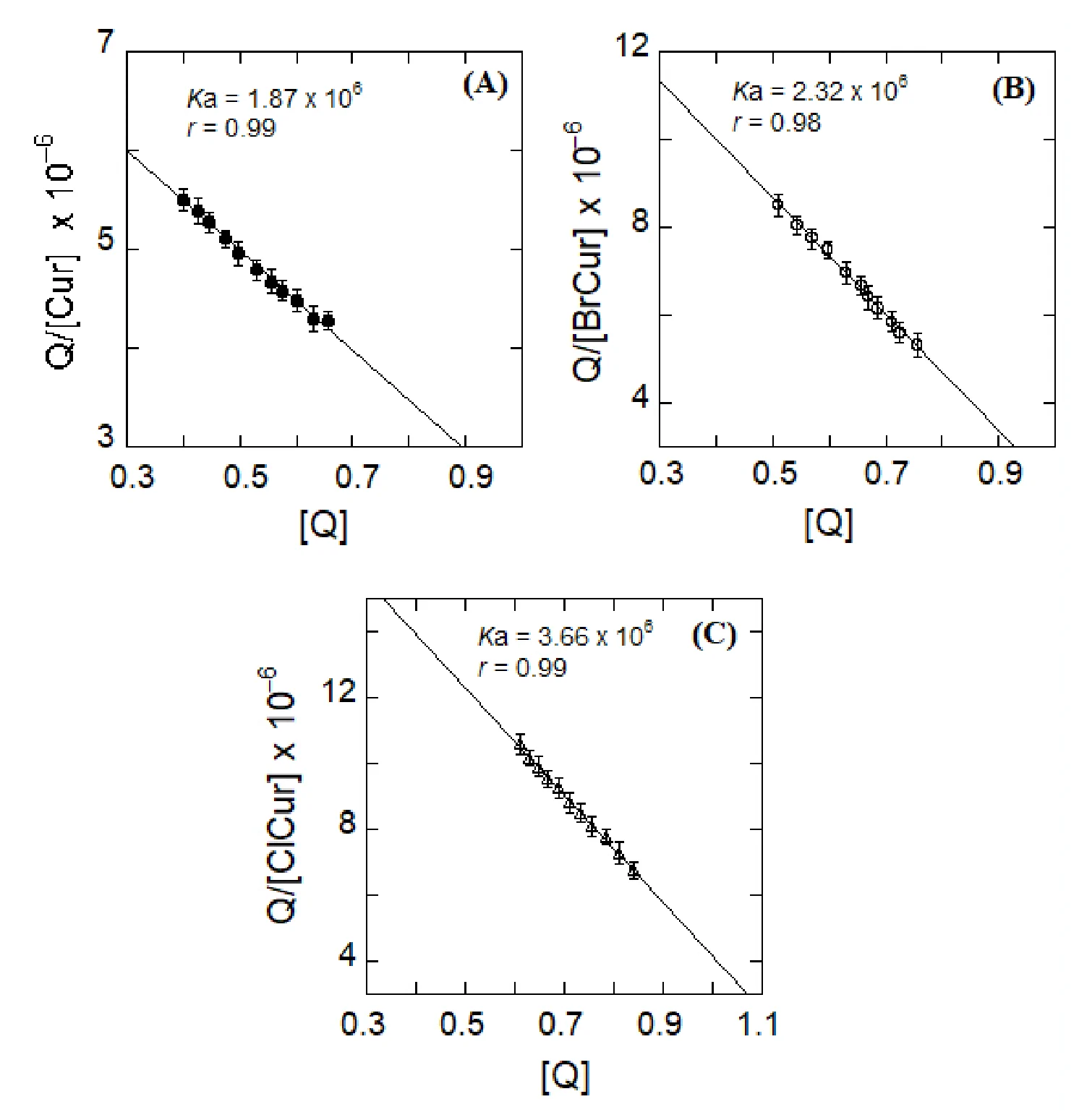
Scatchard analysis of fluorescence quenching titrations for Aβ_1-42_ interacting with (**A**) curcumin, (**B**) α-bromocurcumin, and (**C**) α-chlorocurcumin. Values represent the mean ± SD from three independent experiments.

We further analyzed the binding of the same fluorescence intensity data by fitting them into Scatchard plots. Figure 4 shows the corresponding Scatchard plot for the α-halogenated curcumins binding to Aβ_1-42_. Linear analysis of Q/[curcumin] × 10^−6^ versus Q was used to estimate the association constant (*K*_a_) and binding capacity (*n*) from the slope and intercept. Table 1 summarizes the parameters obtained for the α-halogenated curcumins. Curcumin yielded a *K*_a_ of 1.87 × 10^6^ M^−1^ and an *n* value of 0.89 ± 0.08, whereas α-bromocurcumin gave corresponding values of 2.32 × 10^6^ M^−1^ and 0.92 ± 0.04. For α-chlorocurcumin, *K*_a_ was 3.66 × 10^6^ M^−1^ and *n* was 1.08 ± 0.06. Among the compounds examined, α-chlorocurcumin had the highest estimated association constant, while binding-capacity values were close to unity for all three curcumins.

The binding capacity (*n*) values are reported together with their associated errors to indicate the uncertainty of the fitted parameters. Although a value of (*n*) close to 1 would be consistent with an approximately one-to-one binding stoichiometry, the experimentally determined values showed some deviation from unity. Such deviations may reflect experimental variability and/or limitations of the applied binding model and therefore were interpreted together with the corresponding errors rather than based on the fitted value alone. Amongst the derivatives, α-chlorocurcumin was found to exhibit the strongest affinity followed by α-bromocurcumin, and curcumin, respectively. The association constant values, evaluated by Scatchard analysis, are consistent with nonlinear regression of the experimental binding data dissociation constant (*K*_d_) obtained directly from the fitted binding model. The corresponding association constant (*K*_a_) was calculated as the reciprocal of *K*_d_ (*K*_a_ = 1/*K*_d_). The reliability of the fitted parameters was assessed from the goodness of fit, residual distribution, and associated parameter uncertainties. Nonlinear analysis of the fluorescence data provided values, *K*_d_ = 524 ± 21 nM for Aβ_1-42_/curcumin, 441 ± 16 nM for Aβ_1-42_/α-bromocurcumin, and 285 ± 15 nM for Aβ_1-42_/α-chlorocurcumin, respectively, which are in good agreement with the *K*_d_ value for Aβ_1-42_/α-halogenated curcumins binding as obtained using Scatchard plot.

### 2.2. Temperature Effect on α-Halogenated Curcumin Binding Aβ_1-42_

**Figure 5:**
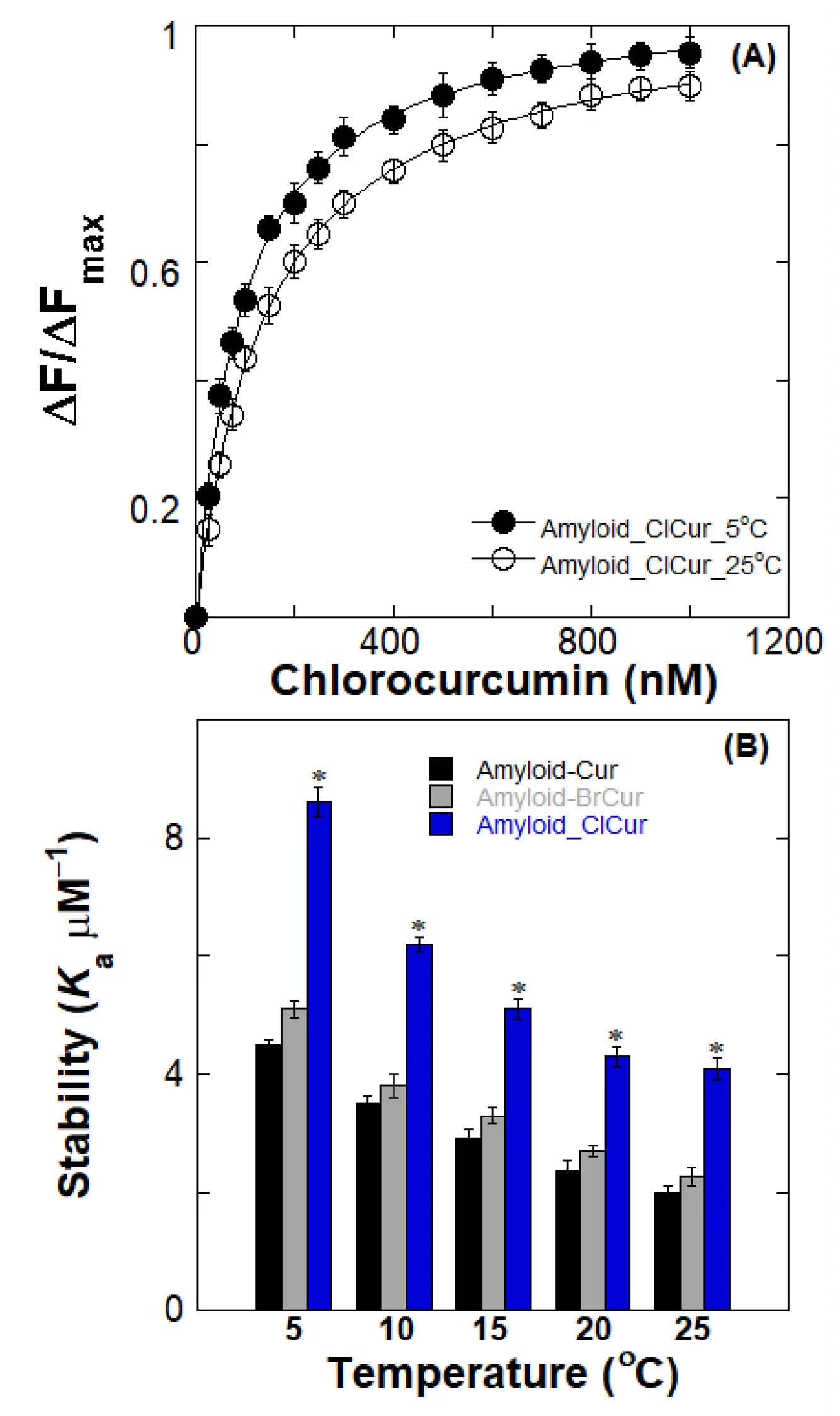
Temperature-dependent binding isotherm for the interaction of Aβ_1-42_ with α-halogenated curcumin. (**A**) A representative binding plot for the titration of Aβ_1-42_ protein (0.1 µM) with varying concentrations of α-chlorocurcumin at 5 °C (―●―) and 25 °C (―○―). (**B**) Bar plot for the binding affinities of Aβ_1-42_ with curcumin and its derivatives at different temperatures. The data are the average of three separate experiments. The values plotted are means of error bars indicate ± SD, and the one-way ANOVA method were used to determine the statistical significance of the differences from Aβ_1-42_/curcumin as compared to α-bromocurcumin and α-chlorocurcumin (* *p* < 0.001) at different temperatures.

The interaction between Aβ_1-42_ and α-halogenated curcumin was examined at different temperatures to characterize its thermal dependence. Changes in fluorescence quenching with temperature were assessed to provide insight into the mechanism underlying the observed fluorescence reduction. Each sample of α-halogenated curcumin/Aβ_1-42_ was pre-incubated for 15 min to attain the studied temperatures at 5, 10, 15, 20, and 25 °C, respectively to form the protein and α-halogenated curcumins complex. Figure 5A depicts the temperature-dependent normalized fluorescence curve obtained upon titration of 0.1 μM Aβ_1-42_ protein with varying concentration (0−1.0 μM) of α-halogenated curcumins binding. Table 2 summarizes the dissociation constant obtained at various temperatures for the Aβ_1-42_/α-halogenated curcumin binding. The dissociation constant increased with temperature, indicating weaker binding of α-halogenated curcumin to Aβ_1-42_ at higher temperatures. This trend is consistent with reduced stability of the α-halogenated curcumins–Aβ peptide complex as a function of temperature.

Because *K*_d_ and *K*_a_ are inversely related, the temperature-dependent changes were interpreted accordingly. An increase in *K*_d_ with increasing temperature indicates a decrease in binding affinity, whereas an increase in *K*_a_ indicates increased affinity. Therefore, the observed increase in *K*_d_ with temperature is consistent with a temperature-dependent reduction in binding affinity. The *K*_d_ for the Aβ_1-42_ binding to α-halogenated curcumin increases with temperature, with *K*_d_ increasing from 253 ± 8.0 nM at 5 °C to 524 ± 21 nM at 25 °C for curcumin, while α-chlorocurcumin increases *K*_d_ from 117.1 ± 8 nM at 5 °C to 285 ± 15 nM at 25 °C (Table 2). Temperature-dependent fluorescence binding data revealed that at all five temperatures, the binding of Aβ_1-42_ with α-halogenated curcumin had significantly higher *K*_a_ as compared to curcumin alone (Figure 5B).

### 2.3. Thermodynamic of α-Halogenated Curcumin Bind Aβ_1-42_

**Figure 6:**
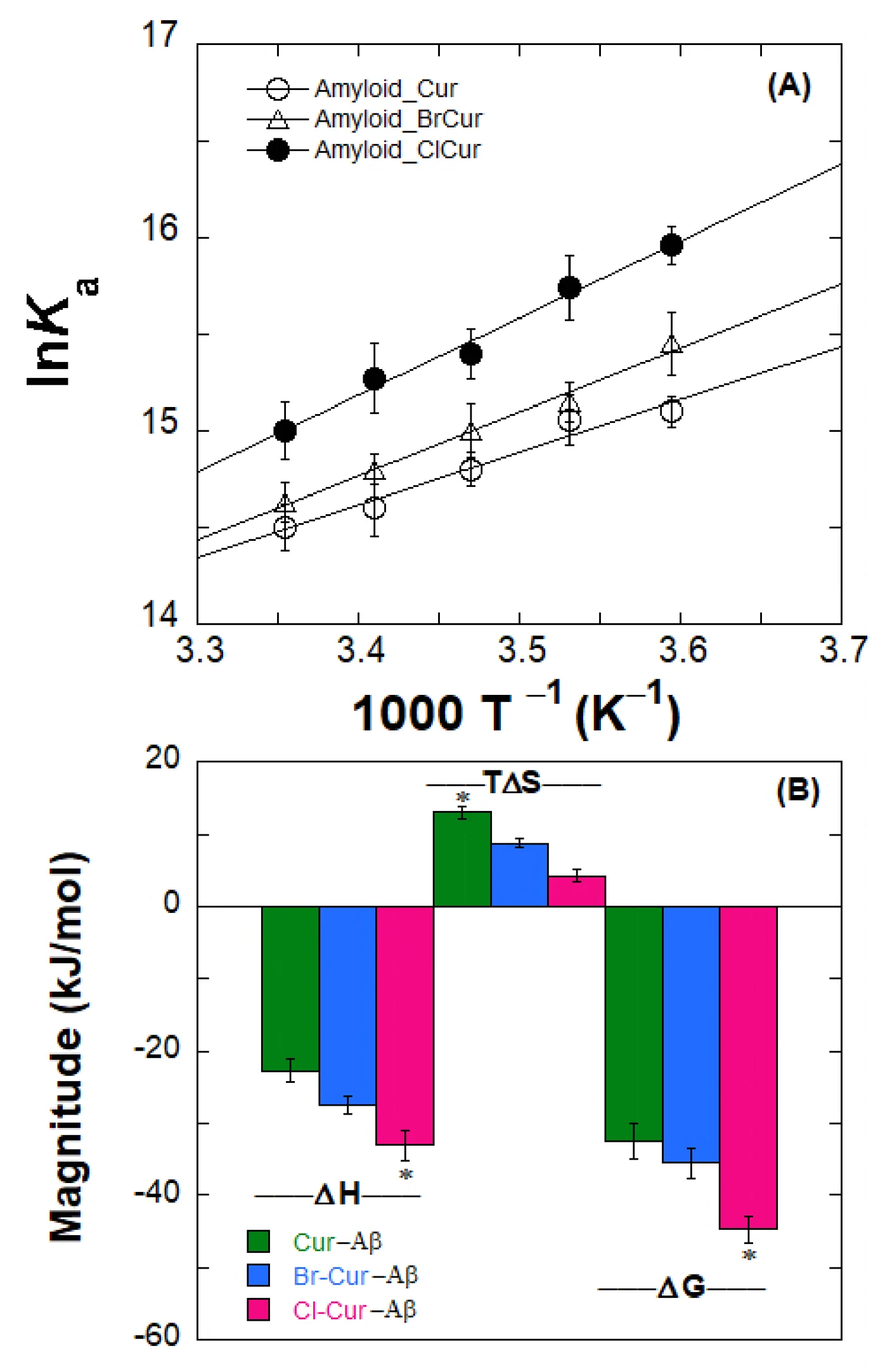
Thermodynamic parameters were determined from the slope and intercept of the Van ’t Hoff plots for the binding of α-halogenated curcumin with Aβ_1-42_ protein. (**A**) Data are indicated as Aβ_1-42_/curcumin (―O―), Aβ_1-42_/α-bromocurcumin (―Δ―), and Aβ_1-42_/α-chlorocurcumin (―●―), respectively. The thermodynamic parameters of α-halogenated curcumin binding to Aβ_1-42_ as determined from the slope and intercept of the Van ’t Hoff plots are shown in (**B**). In the bar plot, the estimated values of ΔG, ΔH, and TΔS at 298 K are shown. The average value of the data is presented, and the data are the average of three separate experiments. The shown data is mean ± SD. The results of the statistical analysis conducted using the one-way ANOVA method for the significance of the differences between curcumin and α-halogenated curcumin (* *p* < 0.001).

Thermodynamic analysis was performed to characterize the energetic contributions to the association of curcumin and its α-halogenated derivatives with Aβ_1-42_. The temperature-dependent association constants (*K*_a_) listed in Table 2 were used to plot ln *K*_a_ against reciprocal absolute temperature (1/*T*) (Figure 6). Linear regression of the Van ’t Hoff plots yielded the enthalpy and entropy changes through the relationships ΔH = −*R* × slope and ΔS = *R* × intercept. Gibbs free energy changes (ΔG) were calculated using Equations (1) and (2), and the resulting thermodynamic parameters are summarized in Table 3.

Curcumin binding exhibited a negative enthalpy change of −20.8 ± 0.7 kJ/mol and a positive entropy change of 43.7 ± 1.4 J/mol/K, indicating favorable contributions from both terms. Halogen substitution shifted this balance toward a greater enthalpic contribution and a smaller favorable entropy contribution. For α-chlorocurcumin, Δ*H* and Δ*S* were −34.0 ± 1.6 kJ/mol and 14.3 ± 0.7 J/mol/K, respectively. The corresponding values for α-bromocurcumin were −27.5 ± 1.1 kJ/mol and 29.4 ± 0.9 J/mol/K. Thus, α-chlorocurcumin showed the most favorable binding enthalpy, whereas unmodified curcumin displayed the largest positive entropy change. The negative Δ*H* and positive Δ*S* values indicate that association was both enthalpically and entropically favorable over the temperatures examined. These findings are compatible with several contributions to binding, including ligand–peptide contacts and changes in solvation.

The Gibbs free energy change (ΔG) provides a thermodynamic measure of the favorability of a binding process. The negative Δ G values obtained for all three compounds indicate that the association of curcumin, α-bromocurcumin, and α-chlorocurcumin with Aβ_1-42_ is thermodynamically favorable under the experimental conditions. The thermodynamic parameters for the interaction of the α-halogenated curcumins with Aβ_1-42_ are presented in Figure 6B. The at ΔG values 298 K were calculated using the thermodynamic parameters obtained from the Van ’t Hoff analysis and Equation 2. The calculated ΔG values were −33.8 ± 0.8, −36.3 ± 1.2, and −38.3 ± 2.0 kJ/mol for curcumin, α-bromocurcumin, and α-chlorocurcumin, respectively. Thus, α-chlorocurcumin exhibited a more favorable binding free energy than curcumin, with a difference of approximately 4.5 kJ/mol. This difference reflects the overall change in the thermodynamic favorability of the association and cannot, by itself, be assigned to the formation of a specific hydrogen bond, salt bridge, or other individual molecular interaction.

The thermodynamic analysis further indicates that the binding of the compounds is influenced by both enthalpic and entropic contributions. For α-chlorocurcumin, the observed changes in ΔH and ΔS relative to curcumin indicate an altered balance between these two contributions following halogenation. The reduced entropic contribution should therefore be considered together with the corresponding enthalpic contribution when interpreting the overall free-energy change. These thermodynamic differences may arise from changes in the physicochemical properties, molecular environment, and conformational or solvation characteristics associated with halogenation; however, the present thermodynamic data alone does not permit identification of the specific molecular interactions responsible for these changes.

The increasingly negative ΔG values from curcumin to α-bromocurcumin and α-chlorocurcumin indicate progressively more favorable overall association with Aβ_1-42_ under the experimental conditions. This finding suggests that halogen substitution influences the thermodynamic properties of curcumin–Aβ_1-42_ association, although the precise molecular basis of this effect cannot be established solely from the thermodynamic measurements. Accordingly, the thermodynamic results are interpreted as evidence of differences in the overall energetics of binding rather than as direct evidence for specific intermolecular contacts. These findings provide complementary information for understanding the binding behavior underlying the observed fluorescence quenching and support the conclusion that α-halogenation modulates the interaction of curcumin derivatives with Aβ_1-42_.

### 2.4. Molecular Docking Analysis

**Figure 7:**
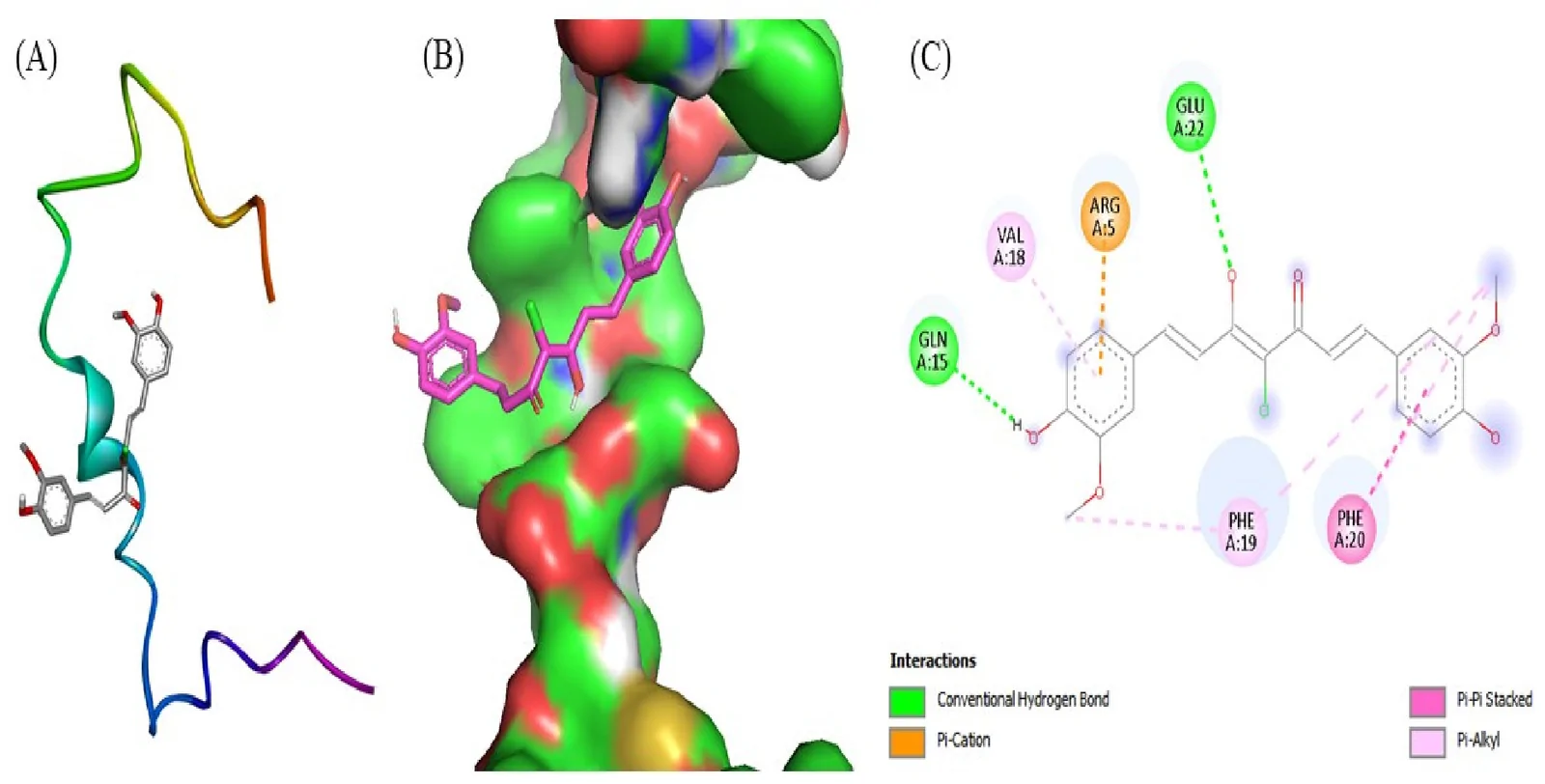
Docking pose of chloro-curcumin with amyloid-beta (**A**) 3D structure showing overall docking of chloro-curcumin with amyloid-beta, and these are represented in the stick model and cartoon diagram, respectively. (**B**) Surface potential view of amyloid beta pocket, along with occupied chloro-curcumin (pink stick) (**C**) 2D representation of chloro-curcumin interaction with the binding pocket residues.

Molecular docking was performed to investigate the predicted binding mode and intermolecular interactions of chloro-curcumin with amyloid-β. Flexible docking yielded a docking score of −5.7 kcal/mol, suggesting an energetically favorable binding pose within the computational model. The predicted complex showed α-chlorocurcumin interacting with several Aβ residues (Figure 7A). A closer examination of the docking pose indicated that the ligand occupied a defined binding region in the modeled peptide structure, positioning its functional groups near residues capable of forming stabilizing noncovalent contacts (Figure 7B).

Analysis of the intermolecular contacts identified two predicted hydrogen bonds involving Glu22 and Gln15 (Figure 7C). These interactions may help anchor chloro-curcumin within the binding region and support its orientation relative to the peptide. Additional π-cation, π–π stacking, and π-alkyl contacts were identified between the ligand and surrounding residues. Together, these interactions suggest that hydrogen bonding and contacts involving the aromatic groups of α-chlorocurcumin contribute to stabilization of the predicted complex. The docking results therefore provide a structural hypothesis for α-chlorocurcumin–Aβ association; however, the docking score represents a computational estimate rather than an experimentally measured binding affinity.

### 2.5. Conformational Analysis α-Halogenated Curcumin Binding to Aβ_1-42_

**Figure 8:**
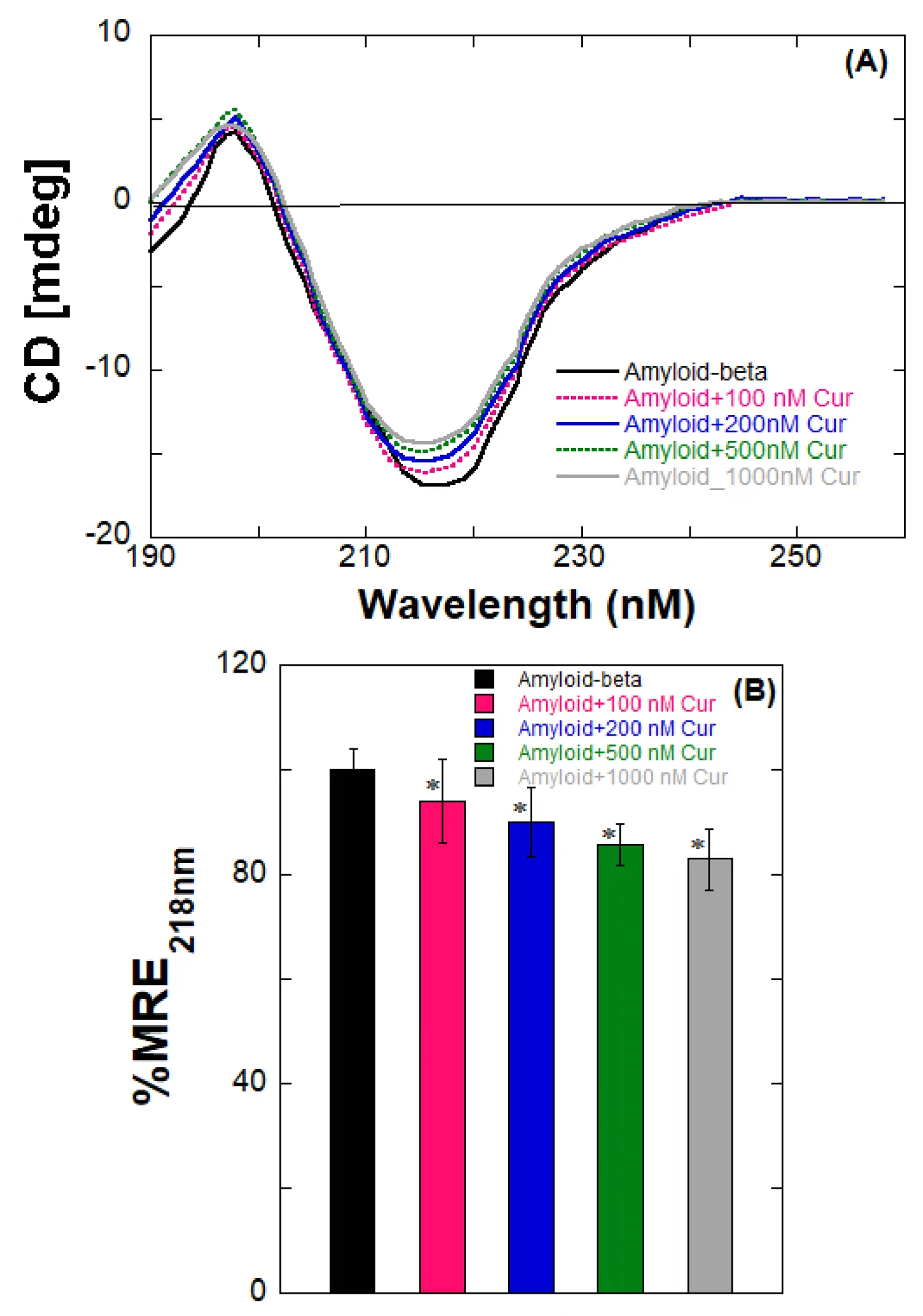
CD spectra and secondary structure determination of Aβ_1-42_ aggregates with curcumin. Ellipticity of free Aβ_1-42_ protein and with addition of increasing concentration of curcumin. (**A**), CD spectra of Aβ_1-42_ (0.1 µM) with adding varying concentrations of curcumin (0–1 µM). (**B**), Bar plot for the quantification of secondary structure measured at spectral minimal mean residual ellipticity at 218 nm. The data is the average of three experiments. Error bars indicate ± SD. The results of the statistical analysis conducted using the one-way ANOVA method for statistical significance of the differences between Aβ_1-42_ alone and in the presence of curcumin (* *p* < 0.02).

Far-UV circular dichroism (CD) spectroscopy was used to examine changes in the secondary structure of Aβ_1-42_ following exposure to curcumin and its α-halogenated derivatives. The spectrum of peptide alone served as the reference for evaluating ligand-associated changes. Figure 8A shows spectra recorded at a fixed Aβ_1-42_ concentration of 0.1 μM with curcumin concentrations ranging from 0.1 to 1.0 μM. In the absence of curcumin, Aβ_1–42_ exhibited a pronounced negative band near 218 nm and a positive band around 195 nm, consistent with a β-sheet-rich conformation. Changes in the far-UV spectral profile upon ligand addition were assessed to characterize alterations in peptide secondary structure. Spectral deconvolution was used to estimate the relative proportions of α-helix, β-sheet, β-turn, and random coil [31,32]. Figure 8B summarizes the normalized mean residue ellipticity signal at 218 nm. Relative to the untreated control (100.00 ± 8.00%), the signal decreased to 94.16 ± 6.60%, 90.00 ± 4.00%, 85.80 ± 5.80%, and 83.00 ± 3.70% at curcumin concentrations of 100, 200, 500, and 1000 nM, respectively (mean ± SE). These values correspond to reductions of 5.84%, 10.00%, 14.20%, and 17.00%, supporting a concentration-dependent attenuation of the β-sheet-associated CD signal.

The α-halogenated curcumin derivatives also altered the far-UV CD spectra in a concentration-dependent manner. Full-spectrum analysis using CDNN suggested changes in the estimated contributions of β-sheet, α-helix, and other secondary-structure components. Because changes in ellipticity at 218 nm do not directly measure β-sheet content, and deconvolution estimates depend on the analytical model and potential ligand contributions, these findings support ligand-associated conformational changes rather than a definitive β-sheet-to-helix conversion.

**Figure 9:**
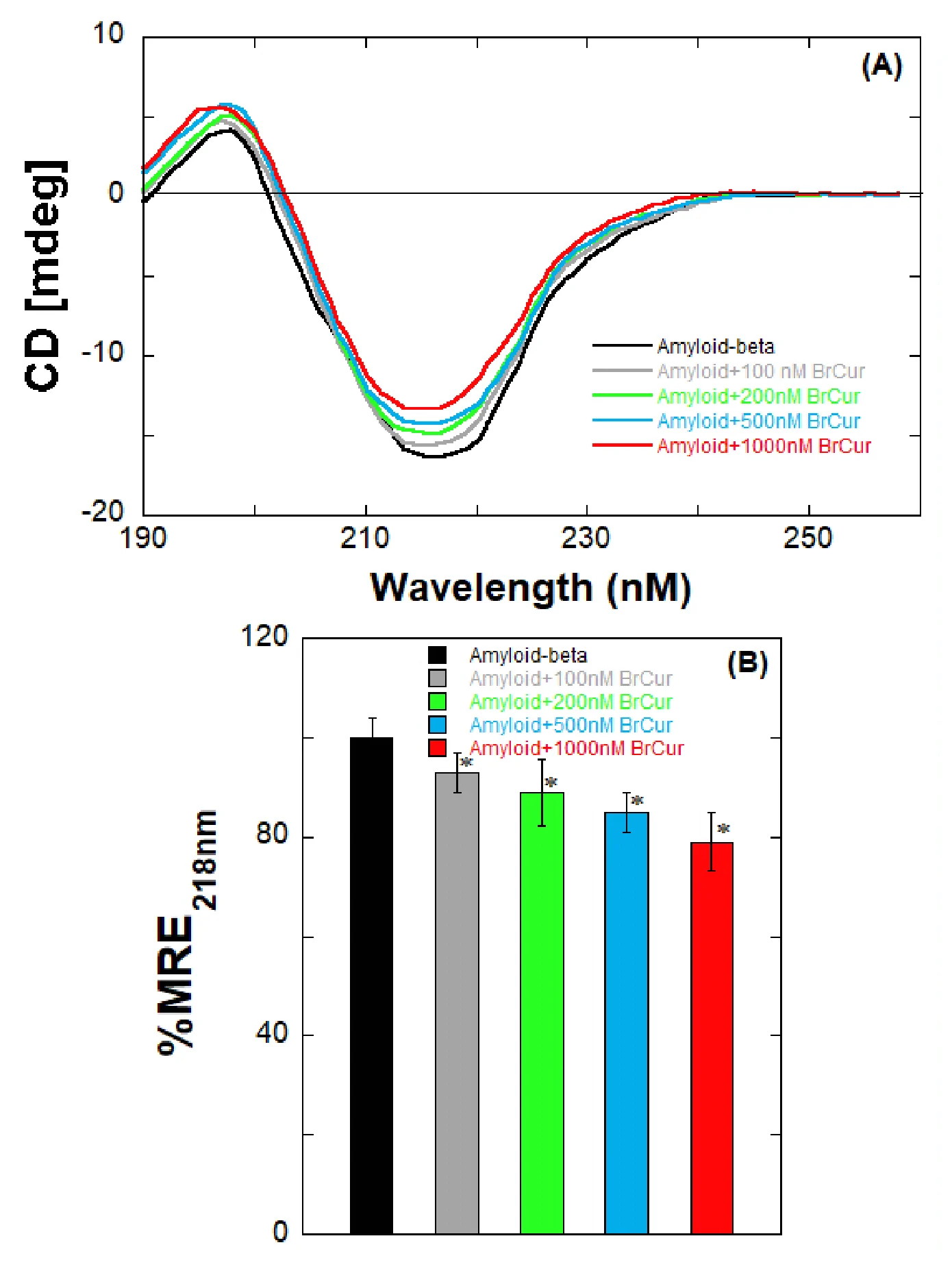
CD spectra and secondary structure determination of Aβ_1-42_ aggregates with α-bromocurcumin. Ellipticity of free Aβ_1-42_ protein and with addition of increasing concentration of α-bromocurcumin. (**A**), CD spectra of Aβ_1-42_ (0.1 µM) adding varying concentrations of α-bromocurcumin (0–1 µM). (**B**), Bar plot for the quantification of secondary structure measured at spectral minimal mean residual ellipticity at 218 nm. The data is the average of three experiments. Error bars indicate ± SD. The results of the statistical analysis conducted using the one-way ANOVA method for statistical significance of the differences between Aβ_1-42_ alone and in the presence of curcumin (* *p* < 0.02).

The effects of α-halogenated curcumin derivatives on Aβ_1–42_ conformation were further examined by far-UV CD spectroscopy. Figure 9A shows the spectra obtained with increasing α-bromocurcumin concentrations. The negative band near 218 nm, associated with β-sheet-rich conformations, progressively weakened over the concentration range of 100–1000 nM. The signal became less negative as ligand concentration increased, indicating a concentration-dependent alteration in the peptide’s CD profile. Figure 9B summarizes the normalized ellipticity response at 218 nm. At 1000 nM α-bromocurcumin, the relative signal decreased to 77% of the peptide-only control, corresponding to a 23% reduction in band magnitude. This attenuation supports changes in β-sheet-associated spectral features but does not directly establish a corresponding decrease in β-sheet content.

**Figure 10:**
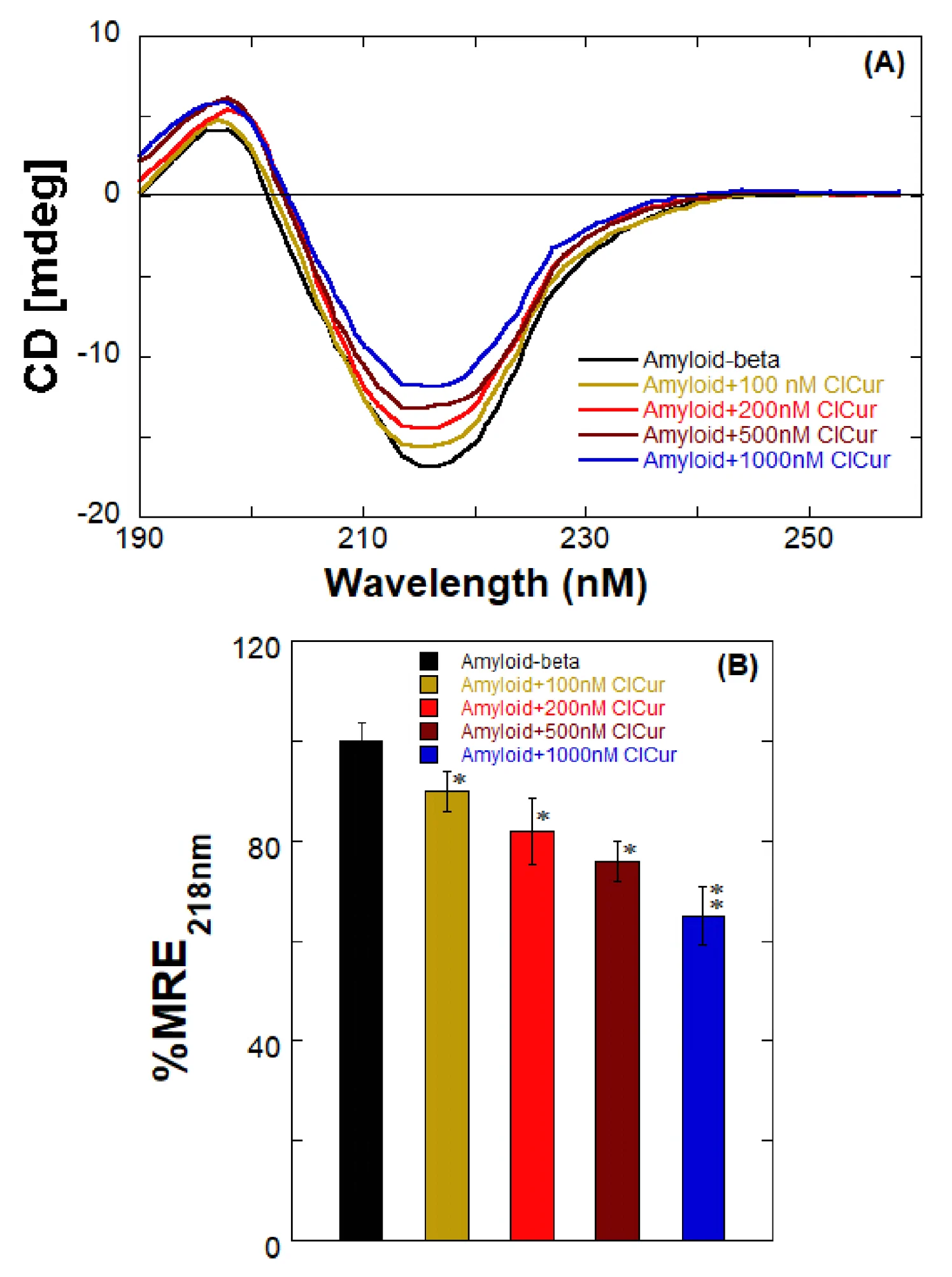
CD spectra and secondary structure determination of Aβ_1-42_ aggregates with α-chlorocurcumin. Ellipticity of free Aβ_1-42_ protein and with addition of increasing concentration of α-chlorocurcumin. (**A**), CD spectra of Aβ_1-42_ (0.1 µM) adding varying concentrations of α-chlorocurcumin (0–1 µM). (**B**), Bar plot for the quantification of secondary structure measured at spectral minimal mean residual ellipticity at 218 nm. The data is the average of three experiments. Error bars indicate ± SD. The results of the statistical analysis conducted using the one-way ANOVA method for statistical significance of the differences between Aβ_1-42_ alone and in the presence of curcumin (* p < 0.02; ** *p* < 001).

Finally, we studied the behavior of Aβ_1-42_ with the addition of chloro-curcumin (Figure 10A). The CD spectra followed the same pattern as mentioned above for the curcumin and α-bromocurcumin. Addition of α-chlorocurcumin further reduced the band intensity of Aβ_1-42_ at 218 nm. Reduction in the band intensity at 218 nm suggests the reduction in the β-sheet secondary structure, which suggests an increase in α-helical content in the protein with the addition of α-chlorocurcumin. Remarkably, the addition of α-chlorocurcumin is linked to significant disturbance in the β-sheet secondary structure of the Aβ_1-42_ protein, which revealed a reduction in the β-sheet secondary structure content from 100% (control, Aβ_1-42_ alone) to 65% at 1000 nM α-chlorocurcumin (Figure 9B). These findings demonstrated that in the presence of α-halogenated curcumin, Aβ_1-42_ protein displayed more structural modification. Following α-halogenated curcumin addition at 1.0 μM, Aβ_1-42_ secondary structure contents directly dropped by 17% for curcumin, 23% for α-bromocurcumin, and 35% for α-chlorocurcumin. These modifications to the secondary structure show how the addition of curcumin derivatives affects specific structural alterations. This significant decrease in the percentage of β-sheet secondary structure was accompanied by a significant increase in the percentage of α-helical content. An increase in α-helical content in the amyloid protein suggests increased stability of the protein, which may lead to inhibition of amyloid-β protein aggregation in Alzheimer’s disease.

### 2.6. Cross-Activity and Fluorescence Response

The cross-reactivity assay evaluated whether the fluorescence enhancement of α-chlorocurcumin observed in the presence of Aβ_1-42_ also occurred with selected other biomolecules. α-Chlorocurcumin (10 μM) was incubated separately with Aβ_1-42_, L-arginine, collagen, gelatin, L-phenylalanine, L-tryptophan, L-tyrosine, and bovine serum albumin at 5 μg/mL concentrations for each comparator. These comparators included free amino acids and non-Aβ proteins to assess potential interference with the fluorescence response. Measurements were performed under identical conditions to those described above for the fluorescence binding experiments. The excitation and emission wavelength of the α-chlorocurcumin were 430 nm and 537 nm, respectively. Fluorescence responses were compared to the -chlorocurcumin-biomolecules complex after the corrected fluorescence intensity of α-chlorocurcumin and biomolecules alone in buffer. Data are presented as average mean ± SD from three independent experiments. The results of the statistical analysis was conducted using the one-way ANOVA method for statistical significance, with the resulting significance indicated as ** *p* < 0.001.

**Figure 11:**
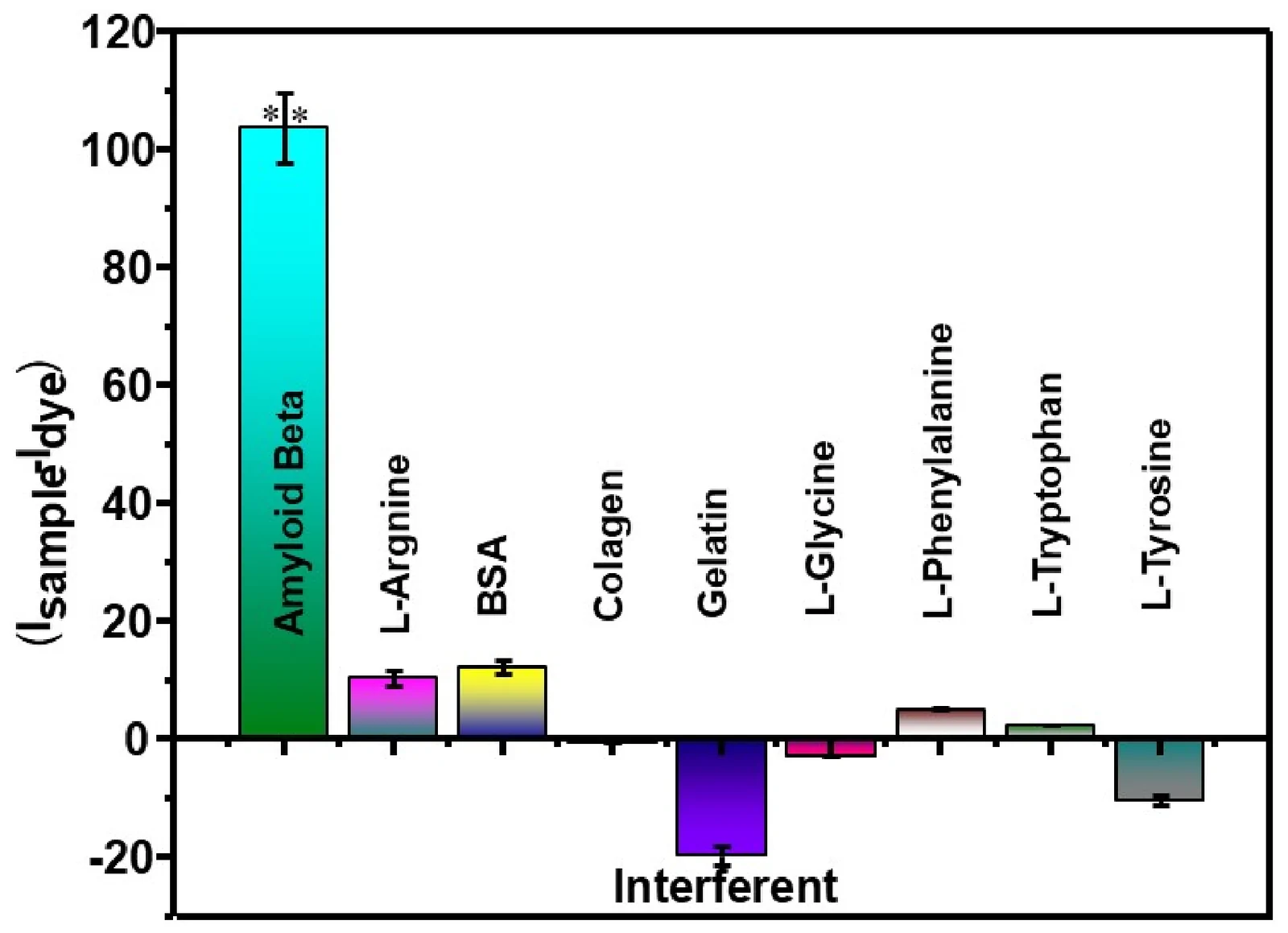
Cross reactivity response as a change in fluorescence intensity (normalized fluorescence) of α-chlorocurcumin versus other molecules. The concentration of α-chlorocurcumin and binding molecules are 10 µM and 5 µg/mL, respectively. The excitation and emission wavelength of the α-chlorocurcumin were 430 nm and 537 nm, respectively. The data is the average of three experiments. Error bars indicate ± SD. The results of the statistical analysis conducted using the one-way ANOVA method for statistical significance of the differences between Aβ_1-42_ and corresponding interfering biological molecules with α-chlorocurcumin (** *p* < 0.001).

As shown in Figure 11, Aβ_1-42_ produced a greater fluorescence enhancement than the selected comparators, most of which showed responses close to that of α-chlorocurcumin alone. These observations support a preferential fluorescence response to Aβ_1-42_ within the tested panel and experimental conditions. However, the panel does not establish exclusive Aβ_1-42_ binding specificity, because related peptides and other amyloid-forming proteins were not evaluated. Moreover, a weak fluorescence response does not necessarily indicate an absence of binding. The observed enhancement may reflect restricted molecular motion or changes in the local environment of α-chlorocurcumin upon association with Aβ_1-42_, consistent with previous reports of curcumin–Aβ interactions [33,34]. Nevertheless, this assay alone does not establish a specific binding site, protein conformational changes, or inhibition of aggregation. These findings therefore indicate preferential fluorescence enhancement with Aβ_1-42_ among the tested biomolecules, rather than definitive molecular specificity.

## 3. Discussion

This study examined the interactions of α-halogenated curcumin derivatives with Aβ_1-42_ using fluorescence spectroscopy, circular dichroism (CD), and molecular docking. Previous studies have reported curcumin binding to Aβ_1-42_ [29,34] and associated changes in peptide conformation, including decreased β-sheet content and increased α-helical content [14]. Modifications to the curcumin scaffold can influence Aβ binding and aggregation, although their effects depend on the substitution pattern. Structure–activity investigations have evaluated curcumin analogs as aggregation inhibitors [35] and examined their interactions with fibrillar aggregates [36]. Lakey-Beitia et al. reported greater anti-aggregation activity for selected aromatic derivatives than for curcumin, demonstrating that improvements can also be achieved through modifications other than α-halogenation [37].

In the present study, α-chlorocurcumin exhibited twofold higher apparent binding affinity than curcumin, with estimated *K*_d_ values of 285 and 524 nM, respectively, whereas α-bromocurcumin showed lower affinity. The binding advantage was therefore specific to α-chlorination rather than a general effect of α-halogenation. These results suggest that the identity of the α-position substituent influences curcumin–Aβ peptide recognition and support further comparative evaluation of these derivatives. Thermodynamic analysis provided insight into the energetic contributions to binding. The Van ’t Hoff plots indicated that curcumin association with Aβ_1-42_ was favored by both enthalpy and entropy, with ΔH = −20.8 ± 0.7 kJ/mol and ΔS = 43.7 ± 1.4 J/mol/K. Compared with curcumin, α-chlorocurcumin exhibited a binding enthalpy approximately 13.2 kJ/mol more negative but a smaller favorable entropy contribution. This shift indicates that α-chlorination alters the balance of thermodynamic contributions to association, consistent with earlier reports [38,39]. Negative ΔG values supported thermodynamically favorable binding under the conditions examined. Differences among the compounds may reflect changes in ligand properties, peptide conformation, and solvent reorganization, including solvation and desolvation.

The phenolic hydroxyl an-d methoxy groups, aromatic rings, and β-diketone unit of curcumin provide potential sites for noncovalent contacts with Aβ_1-42_ [40,41]. Hydrogen bonding, hydrophobic effects, electrostatic and van der Waals interactions, aromatic contacts, and solvent rearrangement may contribute to complex formation [41,42], as suggested by earlier studies of curcumin association with proteins and amyloid-related peptides [43,44]. However, the thermodynamic measurements cannot resolve the contributions of individual forces or independently establish cooperative binding. Molecular docking offers plausible binding poses and potential residue contacts, but these predictions require validation using complementary structural methods. CD measurements revealed ligand-associated changes in the conformational characteristics of Aβ_1-42_. At a ligand concentration of 1 μM, α-bromocurcumin and α-chlorocurcumin reduced the magnitude of peptide ellipticity at 218 nm by 23% and 35%, respectively. The larger response to α-chlorocurcumin indicates a greater spectral change under these conditions. Nevertheless, changes in CD intensity do not directly quantify the proportions of individual secondary-structure components.

Together, the fluorescence and CD findings support curcuminoid–Aβ_1-42_ association and accompanying conformational changes, but do not establish inhibition of oligomerization, fibril formation, or peptide toxicity. Studies combining binding measurements with aggregation assays, including investigations of arene–ruthenium (II) curcumin derivatives [45], provide a useful framework for further evaluation. Thioflavin T aggregation kinetics, morphological analysis by transmission electron microscopy or atomic force microscopy, and toxicity assays under matched conditions are needed to determine whether the binding advantage of α-chlorocurcumin translates into improved anti-amyloid activity.

## 4. Materials and Methods

### 4.1. Materials

Curcumin and Aβ_1-42_ protein were obtained from MOLEQULE-ON (New Lynn, Auckland, New Zealand). α-halogenated derivatives, α-bromocurcumin and α-chlorocurcumin, were synthesized and purified as described previously [27,28]. Double distilled, deionized water from a Milli-Q purification system, with a resistivity of 18.2 MΩ·cm, was used for all buffer preparation. Other reagents were of molecular biology grade.

### 4.2. Preparation of Stock Solution

Curcumin, α-bromocurcumin, and α-chlorocurcumin were dissolved separately in dimethyl sulfoxide (DMSO) to prepare stock solutions of 50 mM. Solutions were prepared immediately before use. Working solutions were prepared by diluting stocks into 20 mM HEPES/KOH buffer, pH 7.4, to obtain final ligand concentrations of 0–1.0 µM. To keep Aβ1-42 in the monomer form the lyophilized Aβ_1-42_ peptide (10 mg) was dissolved in one milliliter Hexafluoro isopropanol (HFIP) to completely break up any pre-existing aggregates. Aliquot the solution into microcentrifuge tubes and let the HFIP evaporate completely to form a clear peptide film and store at −20 °C until used. Resuspend the peptide in an ice cold DMSO. Dilute the stock solution directly into 20 mM HEPES/KOH buffer, pH 7.4 immediately before running the experiments. The concentration of protein was estimated by Bio-Rad protein assay reagent following the standard Bradford method [46].

### 4.3. Fluorescence Titration Measurements

Fluorescence measurements were performed using an FP-8550 spectrofluorometer (JASCO, Tokyo, Japan) coupled with a data recorder and a temperature controller. Fluorescence titrations were performed in 20 mM HEPES/KOH buffer, pH 7.4 containing 0.1 µM Aβ_1-42_ protein concentration. The interaction of Aβ_1-42_- α-halogenated curcumins were measured by direct protein fluorescence. Intrinsic fluorescence emission spectra of the native protein were recorded to monitor changes in fluorescence intensity and emission profile upon interaction with the α-halogenated curcumins. Samples were excited at 280 nm, and emission was measured from 300 to 400 nm using excitation and emission slit widths of 5 and 10 nm, respectively. Measurements were conducted in a cuvette with a 1 cm optical path length. The excitation slit width was selected to minimize photobleaching while maintaining sufficient fluorescence intensity. The absorbance of the samples at the excitation wavelength was maintained below 0.02 throughout the fluorescence measurements. Because the absorbance at the excitation wavelength was very low, attenuation of the excitation and emitted light within the sample was negligible. Therefore, the inner filter effect was considered minimal, and no mathematical inner-filter correction was applied to the fluorescence data. The low absorbance condition was maintained for all samples to ensure that the measured fluorescence changes primarily reflected ligand-dependent fluorescence quenching rather than optical attenuation. Before fluorescence measurements, all samples were equilibrated for at least 15 min. A fixed concentration of Aβ_1-42_ (0.1 µM) was incubated with increasing concentrations of the α-halogenated curcumin derivatives (0–1.0 µM) to obtain the desired molar ratios. The final volume of each sample was adjusted to 1 mL with 20 mM HEPES/KOH buffer (pH 7.4). Binding affinity was evaluated by monitoring the changes in Aβ_1-42_ intrinsic fluorescence upon titration with the α-halogenated curcumin derivatives. The resulting fluorescence data were analyzed by nonlinear regression to determine the binding parameters [47]. Fluorescence intensity at the emission maximum (332 nm) was used to quantify changes in the intrinsic fluorescence of Aβ_1-42_, with the intensity of the protein-only control defined as 100%. To account for the individual contributions of the protein and ligand, fluorescence was measured separately for Aβ_1-42_ and the α-halogenated curcumin derivative at the corresponding concentrations. The corrected intensities from these individual samples were added to obtain the summed fluorescence signal (Fs). Subsequently, Aβ_1-42_ was mixed with the same concentration of the ligand, and the corrected fluorescence intensity of the resulting mixture was recorded (F_c_). The fluorescence difference associated with complex formation was calculated as ΔF = F_s_ − F_c_. The normalized fluorescence difference ΔF/ΔF_max_ was then used to estimate the dissociation constant (*K*_d_) according to the specified binding model [48,49]. Measured fluorescence intensities of Aβ_1-42_/α-halogenated curcumin complexes were corrected by subtracting fluorescence of α-halogenated curcumin alone in buffer. The average value of three separate titration trials was provided for each equilibrium measurement. Nonlinear least squares fitting of the corrected data used to determine the *K*_d_ values using KaleidaGraph version 2.1.3 (Abelbeck Software, Reading, PA, USA).

The selectivity of α-chlorocurcumin for Aβ_1-42_ was evaluated by assessing its fluorescence response in the presence of potential interfering biomolecules. Under identical experimental conditions, α-chlorocurcumin (1 µM) incubated separately with Aβ_1-42_ (1 µM) and with each potential interferent at the corresponding concentration. Following incubation, fluorescence emission measured at 537 nm using an excitation wavelength of 430 nm. Fluorescence enhancement was assessed by comparing the signal obtained for α-chlorocurcumin in the presence of Aβ_1-42_ with those recorded in the presence of the other biomolecules. The relative fluorescence responses were presented as a bar graph to illustrate the selectivity profile of α-chlorocurcumin. Fluorescence data were processed and plotted using KaleidaGraph (version 2.1.3; Abelbeck Software). All measurements were conducted in triplicate, and the results are reported as mean ± standard deviation (SD), with error bars representing the variability among the three measurements.

### 4.4. Determination of Binding Site

Binding of Aβ_1-42_ to different α-halogenated curcumins were studied using fluorescence titration experiments. Fluorescence quenching titration of Aβ_1-42_ protein with increasing concentrations of α-halogenated curcumin derivatives (α-bromocurcumin and α-chlorocurcumin) were performed in a discontinuous manner. Samples containing 0.1 μM Aβ_1-42_ were prepared with progressively increasing amounts of α-halogenated curcumins from a 50 mM stock solution, giving ligand-to-peptide molar ratios of 0–10. Each mixture was adjusted to a final volume of 1 mL using 20 mM HEPES/KOH buffer (pH 7.4) and incubated at room temperature for 15 min before measurement. Fluorescence emission recorded over 300–400 nm following excitation at 280 nm. The decrease in Aβ_1–42_ fluorescence upon ligand addition was evaluated relative to the peptide-only control. The fluorescence intensity of the untreated protein is 100%. The normalized data were analyzed by fitting them in Scatchard plots ([50] as described earlier [51] using the following equation: n*K*_a_ − *K*_a_Q = Q/[C] = Q/[R − Q][Aβ_1-42_]_T_, where *n* is the binding capacity; *K*_a_, the association constant; Q, the fractional quench; [C] is the free concentration of α-halogenated curcumin; [Aβ_1-42_]_T_ is the total protein concentration and R is the α-halogenated curcumin/Aβ molar ratio. The values of *K*_a_ were obtained from the slope of a straight-line plot between Q[Cur] versus Q, while the *x*-axis of the straight-line plot was used to determine the stoichiometry (average number of binding sites, *n*).

### 4.5. Effect of Temperature on α-Halogenated Curcumins Binding to Aβ_1-42_

The influence of temperature on the binding of α-halogenated curcumin derivatives to Aβ_1-42_ was examined at 5, 10, 15, 20, and 25 °C using the fluorescence protocol described above. At each temperature, 1 mL samples were equilibrated for 15 min before spectra were recorded. A thermostatic controller maintained the sample temperature within ± 0.1 °C of the selected value throughout the measurements.

### 4.6. Thermodynamic Analyses of α-Halogenated Curcumins Binding to Aβ_1-42_

Temperature-dependence equilibrium binding data were used to estimate the binding parameters for the interaction between Aβ_1-42_ and α-halogenated curcumins complex as described elsewhere [52]. Equilibrium binding constants measured at different temperatures were analyzed using a Van ’t Hoff plot to estimate the thermodynamic parameters governing the association of α-halogenated curcumins with Aβ_1-42_.

The enthalpy (ΔH) and entropy (ΔS) changes associated with α-halogenated curcumin binding to Aβ_1-42_ were estimated by fitting ln K_a_ against reciprocal absolute temperature (1/T) using the Van ’t Hoff relationship:

(1) $$\ln\;K_{a\;}=\;-\frac{∆H}{R\;T\;}+\;\frac{∆S}{R}$$

In this equation, *K*_a_ denotes the equilibrium association constant, *T* is the absolute temperature in kelvin, and *R* is the universal gas constant (8.31 J/mol/K). The enthalpy and entropy changes were calculated from the slope and intercept of the ln *K*_a_ *versus* 1/*T* plot, respectively, using Δ*H* = −*R* × slope and Δ*S* = *R* × intercept. The Gibbs free energy change (Δ*G*) for binding was subsequently calculated using Equation (2).

(2) ∆*G*= ∆*H* − *T*∆*S*

### 4.7. Molecular Docking

Molecular docking was performed to investigate potential binding orientations and interactions between Aβ_1-42_ and α-chlorocurcumin. The solution structure of the Aβ protein structure (PDBID:6SZF) [53] was downloaded from the RCSB Proten Data Bank and prepared by adding hydrogen atoms, assigning charges, and defining appropriate atom types. Curcumin structure was retrieved from PubChem, and chlorine was added at the fifth position using Ketcher v.3.12.0. Docking was performed using AutoDock Vina, v1.2.7 [54]. A blind docking approach was used with a docking grid of 40 Å × 40 Å × 52 Å, covering the entire amyloid-beta protein, to identify flexible docking sites. Flexible docking was performed in this docking grid, with the exhaustive parameter set adjusted to nine for thorough conformational sampling. Docking simulations were performed using default parameters, which rank poses by binding affinity and interaction energy. The top docking pose with the highest docking score was selected for visualization. PyMOL (version 2.5.8) was used for visualization, and Discovery Studio was used to draw a 2D interaction diagram.

### 4.8. Circular Dichroism (CD) Measurements

The effects of α-halogenated curcumins: α-bromocurcumin and α-chlorocurcumin on the secondary structure of Aβ_1–42_ proteins were examined by far-UV circular dichroism (CD) spectroscopy. Spectra were collected from 190 to 260 nm using a Chirascan Plus spectropolarimeter (Applied Photophysics Ltd., Surrey, UK) and cuvettes with a 1 mm optical path length. The sample temperature was maintained at 25 °C using a temperature controller connected to a circulating water bath, while continuous nitrogen flow was supplied during spectral acquisition. Before measurements, the instrument was calibrated with D-10-camphorsulphonic acid according to the manufacturer’s instructions. All CD spectra samples were acquired at constant concentration (0.1 µM) of Aβ_1-42_ protein as previously reported [14,47] with varying concentrations (0–1 µM) of curcumin and α-halogenated curcumins and Aβ/α-halogenated curcumins, with a molar ratio of 1:10. Each sample was incubated for 15 min to equilibrate for the complex formation. All samples were filtered before measurement to remove suspended particles. Far-UV CD spectra were recorded over 190–260 nm at a scan rate of 50 nm min^−1^, with a response time of 1 s. Measurements were performed in triplicate, and the mean and standard deviation were calculated. Background correction used the appropriate reference spectrum: buffer alone for peptide-only samples and the corresponding α-halogenated curcumin solution in buffer for ligand-containing samples. The corrected spectra were smoothed using a Savitzky–Golay (SG) filter with a bandwidth setting of 60 and expressed as mean residue ellipticity ([θ], deg cm^2^ dmol^−1^) against wavelength (nm). CDNN 2.1 software was used to estimate the proportions of α-helix, β-sheet, β-turn, and random coil in Aβ samples at different α-halogenated curcumin concentrations.

CD spectra of Aβ_1-42_ in the absence and presence of the α-halogenated curcumin were recorded over the far-UV wavelength range after correction for the corresponding buffer and compound background spectra. Secondary-structure composition was estimated from the full CD spectra using CDNN deconvolution, rather than from ellipticity at a single wavelength. The ellipticity at 218 nm was used as a comparative spectral parameter to monitor changes in the CD response and was not independently converted into β-sheet or α-helical percentages. Compound-only spectra were recorded at the corresponding concentrations and subtracted from the peptide-containing spectra to minimize contributions arising from the intrinsic optical properties of the chromophoric compounds. The resulting secondary-structure estimates were interpreted as model-dependent estimates of conformational composition rather than direct evidence of a specific β-sheet to α-helix conversion.

### 4.9. Statistical Analysis

Quantitative data are reported as mean ± standard deviation (SD) from independent replicate measurements, with error bars representing SD. Fluorescence binding experiments were performed in triplicate. Concentration-dependent fluorescence data were fitted by nonlinear least-squares regression to estimate the apparent dissociation constant (*K*_d_). Differences in independently fitted *K*_d_ values among curcumin derivatives were evaluated using one-way analysis of variance (ANOVA). The standard error (SE) of each fitted parameter was calculated as the square root of the corresponding diagonal element of the regression covariance matrix. Goodness of fit was assessed using the coefficient of determination (*R*^2^) and root-mean-square error (RMSE).

Far-UV circular dichroism (CD) spectra of Aβ_1-42_ in the absence and presence of ligands were recorded in triplicate under identical experimental conditions. CD signals were expressed as mean residue ellipticity (MRE), and spectra were deconvoluted using the CONTIN algorithm available on the DICHROWEB platform [55]. Secondary-structure percentages obtained from individual replicate spectra were summarized as mean ± SD. Treatment effects were evaluated using one-way ANOVA followed by Dunnett’s post hoc test, with the Aβ_1-42_-only group serving as the control. Statistical significance was defined as *p* < 0.05.

## 5. Conclusions

Fluorescence spectroscopy, circular dichroism spectroscopy, and molecular docking were used to investigate the interactions of α-halogenated curcumin derivatives with Aβ_1-42_. Temperature-dependent fluorescence measurements indicated differential binding and yielded apparent binding constants and binding stoichiometries. Negative ΔG values derived from thermodynamic analysis were consistent with favorable association under the experimental conditions. Changes in far-UV CD spectra suggest alterations in peptide secondary structure, although these changes alone do not establish complex formation or inhibition of aggregation. Molecular docking predicted possible bound conformations and residue interactions for α-chlorocurcumin; an appreciable binding between α-chlorocurcumin and Aβ_1-42_ was observed. Molecular docking results clearly showed that α-chlorocurcumin occupies the binding pockets with appreciable binding energy and that it forms several close interactions with critically important residues.

Overall, the findings support differential binding and measurable conformational changes in Aβ_1-42_ associated with the α-halogenated curcumins. The observed changes in binding and peptide conformation suggest that these derivatives may modulate the structural behavior of Aβ_1-42_ and potentially influence its aggregation propensity. However, the present findings do not provide direct evidence for inhibition of Aβ_1-42_ oligomerization or fibril formation. Future studies employing direct aggregation-specific approaches, including ThT fluorescence kinetics and morphological analyses using TEM or AFM, are required to determine whether these compounds alter the kinetics, extent, or morphology of Aβ_1-42_ aggregation. Overall, the findings provide a basis for further investigation of α-halogenated curcumin derivatives as potential modulators of Aβ_1-42_ structure and aggregation-related processes.

## Figures and Tables

**Table 1:** Binding parameters obtained for the interaction of Aβ_1-42_ with α-halogenated curcumin derivatives at 25 °C.

Complex	Association Constant *K*_a_ (M^−1^)	Dissociation Constant *K*_d_ (nM)	Binding Capacity (*n*)
Aβ_1-42_/curcumin	1.87 × 10^6^	524 ± 21	0.89 ± 0.08
Aβ_1-42_/α-bromocurcumin	2.32 × 10^6^	441 ± 16	0.92 ± 0.04
Aβ_1-42_/α-chlorocurcumin	3.66 × 10^6^	285 ± 15	1.08 ± 0.06

**Table 2:** Temperature dependent dissociation constant for the interaction between Aβ_1-42_ and α-halogenated curcumin derivatives.

Complex	*K*_d_ (nM)				
	5 °C	10 °C	15 °C	20 °C	25 °C
Aβ_1-42_/curcumin	253 ± 8	287.0 ± 9	347.5 ± 12.0	426 ± 15	524 ± 21
Aβ_1-42_/α-bromocurcumin	195 ± 9	263.6 ± 11	301.3 ± 14	373 ± 12	441 ± 16
Aβ_1-42_/α-chlorocurcumin	117.1 ± 8	153.0 ± 9	196.3 ± 13.0	231.0 ± 14	285.0 ±15

**Table 3:** Thermodynamic parameters for the interaction of Aβ_1-42_ with α-halogenated curcumin derivatives.

Complex	∆*H* kJ/mol	∆*S* J/mol/K	∆*G* kJ/mol	TΔS at 298 K kJ/mol
Aβ_1-42_/curcumin	−20.8 ± 0.7	43.7 ± 1.4	−33.8 ± 0.8	13.0
Aβ_1-42_/α-bromocurcumin	−27.5 ± 1.1	29.4 ± 0.9	−36.3 ± 1.2	8.76
Aβ_1-42_/α-chlorocurcumin	−34.0 ± 1.6	14.3 ± 0.7	−38.3 ± 2.0	4.26

## Data Availability

The corresponding author can provide the datasets used and/or analyzed in this study upon reasonable request.
